# Pulsed and cooled radiofrequency for chronic shoulder pain: a systematic review of comparative and cohort studies

**DOI:** 10.3389/fmed.2026.1747949

**Published:** 2026-08-21

**Authors:** Hassan A. Moria

**Affiliations:** Department of surgery, Faculty of medicine, University of Tabuk, Tabuk, Saudi Arabia

**Keywords:** chronic shoulder pain, cooled radiofrequency ablation, corticosteroid injection, pulsed radiofrequency, suprascapular nerve, systematic review

## Abstract

**Background:**

Chronic shoulder pain is a common cause of pain and disability, and repeated corticosteroid injections often provide only temporary symptom relief. Pulsed radiofrequency (PRF) and cooled radiofrequency ablation (CRFA) have emerged as minimally invasive interventional options; however, their comparative effectiveness and the certainty of the supporting evidence remain uncertain.

**Methods:**

This systematic review was conducted and reported in accordance with the Preferred Reporting Items for Systematic Reviews and Meta-Analyses (PRISMA) 2020 statement. MEDLINE, Embase, the Cochrane Central Register of Controlled Trials (CENTRAL), Scopus, and Web of Science were systematically searched and supplemented by backward citation screening. Randomized and observational clinical studies evaluating PRF or CRFA for chronic shoulder pain were included. Owing to substantial clinical and methodological heterogeneity, the findings were synthesized narratively. Methodological quality and risk of bias were assessed using the Cochrane Risk of Bias 2 (RoB 2) tool, the Risk Of Bias In Non-randomized Studies of Interventions (ROBINS-I) tool, and the Joanna Briggs Institute (JBI) Critical Appraisal Checklist for Case Series. The certainty of evidence was evaluated using the Grading of Recommendations, Assessment, Development and Evaluation (GRADE) approach.

**Results:**

In total, 10 studies involving 476 participants met the eligibility criteria, including five randomized controlled trials, one retrospective comparative study, and four uncontrolled CRFA cohort studies. A total of four randomized trials (*n* = 150) comparing PRF with corticosteroid-based injections or active nerve block interventions consistently demonstrated improvements in pain intensity and shoulder function following PRF. However, PRF did not demonstrate superiority over corticosteroid-based interventions, which generally provided greater short-term pain relief. Furthermore, two studies (*n* = 220) evaluating adjunctive PRF reported more favorable clinical outcomes when PRF was combined with corticosteroid-based treatment than when corticosteroid-based treatment was administered alone. In addition, four uncontrolled CRFA cohort studies (*n* = 106) consistently reported improvements in pain intensity and shoulder function over 6–12 months of follow-up; however, no randomized or controlled comparative studies evaluating CRFA were identified. The certainty of evidence was rated as low for comparative and adjunctive PRF studies and as very low for CRFA studies.

**Conclusion:**

PRF consistently improved pain intensity and shoulder function but did not demonstrate superiority over corticosteroid-based interventions in randomized comparative studies. Preliminary evidence suggests that adjunctive PRF may enhance clinical benefit when combined with corticosteroid-based treatment, whereas evidence supporting CRFA is currently limited to uncontrolled observational studies. Current evidence does not establish a preferred radiofrequency strategy for chronic shoulder pain. Adequately powered, multicenter randomized controlled trials directly comparing PRF, CRFA, and corticosteroid-based interventions are required before definitive treatment recommendations can be made.

**Systematic review registration:**

https://www.crd.york.ac.uk/PROSPERO/view/CRD42026129, identifier CRD420261295837.

## 1 Introduction

Chronic shoulder pain is among the most common musculoskeletal disorders encountered in primary care, orthopedic practice, rehabilitation, and pain medicine. It is associated with persistent pain, upper-limb disability, sleep disturbance, reduced work productivity, impaired quality of life, and increased healthcare utilization. Population-based studies estimate a prevalence ranging from approximately 7% to 27%, although estimates vary according to study population and diagnostic criteria. Although many patients improve with conservative management, symptoms persist beyond 3 months in a substantial proportion, prompting consideration of additional treatment options when pain and functional limitation remain despite nonoperative care (1, 2).

Chronic shoulder pain encompasses a heterogeneous group of conditions rather than a single clinical entity. Rotator cuff-related shoulder pain, adhesive capsulitis, glenohumeral osteoarthritis, and other periarticular disorders account for most chronic presentations encountered in clinical practice. Although these conditions differ in their underlying pathology, they commonly present with persistent pain, restricted range of motion, muscle weakness, and functional limitation. Structural abnormalities identified on imaging do not consistently correlate with symptom severity, indicating that patients with similar clinical presentations may have different underlying pain mechanisms and may respond differently to the same intervention (3, 4).

The current management of chronic shoulder pain generally involves a multimodal approach that may include patient education, activity modification, structured rehabilitation, and pharmacological treatment according to the underlying condition, symptom severity, and individual patient factors. Among interventional options, image-guided corticosteroid injections are commonly used when symptoms persist despite conservative management or limit participation in rehabilitation. Corticosteroid injections have been associated with short-term improvements in pain and function in selected shoulder conditions; however, treatment effects may diminish over time. Repeated corticosteroid injections have also raised concerns regarding tendon integrity, articular cartilage, and cumulative systemic corticosteroid exposure, particularly when administered frequently (5, 6).

Persistent shoulder pain is believed to involve both peripheral and central nociceptive mechanisms in addition to structural pathology. Peripheral nociceptive input may arise from inflammation, tendon degeneration, capsular fibrosis, or mechanical loading, while prolonged nociceptive signaling has been associated with peripheral sensitization and, in some individuals, features of central sensitization. These neurophysiological mechanisms may contribute to persistent pain and functional limitation that are not fully explained by structural abnormalities alone. Recognition of the multifactorial nature of chronic shoulder pain has increased interest in treatment strategies that target nociceptive processing alongside management of the underlying structural condition (7, 8).

The rationale for radiofrequency interventions is informed by the sensory innervation of the shoulder joint. Nociceptive input from the glenohumeral joint is transmitted predominantly through the suprascapular, axillary, and lateral pectoral nerves, with additional contributions from smaller articular branches. Anatomical cadaveric studies have demonstrated an overlap among these sensory pathways, indicating that multiple nerves may contribute to nociceptive transmission from the shoulder joint. These anatomical findings have informed the development of image-guided procedures targeting the principal articular branches while seeking to preserve adjacent motor nerves and surrounding vascular structures (9, 10).

Radiofrequency-based procedures have been increasingly evaluated as interventional options for selected patients with chronic shoulder pain that persists despite appropriate conservative management. Pulsed radiofrequency (PRF) and cooled radiofrequency ablation (CRFA) both apply radiofrequency energy adjacent to sensory nerves but differ in their procedural characteristics. PRF delivers intermittent electrical pulses while maintaining tissue temperatures below those typically associated with thermal neurodestruction and has been proposed to modulate nociceptive signaling. In contrast, CRFA combines radiofrequency energy with internal electrode cooling to produce larger ablation zones than conventional thermal radiofrequency techniques, which may improve lesion coverage in the setting of anatomical variation. These procedural differences have prompted the increasing evaluation of radiofrequency-based interventions as alternatives to repeated corticosteroid injections for selected patients with chronic shoulder pain (11, 12).

Despite increasing clinical use, the evidence supporting radiofrequency interventions for chronic shoulder pain remains limited by heterogeneous patient populations, underlying diagnoses, procedural techniques, comparator interventions, and outcome assessment. Furthermore, PRF has been investigated in randomized comparative trials, whereas evidence supporting CRFA is derived almost exclusively from uncontrolled cohort studies, making direct interpretation of the literature challenging. This systematic review therefore evaluated the effectiveness and safety of PRF and CRFA by separately synthesizing comparative PRF studies, adjunctive PRF studies, and uncontrolled CRFA cohort studies. In addition, the review assessed methodological quality and the certainty of evidence and identified priorities for future research (11, 13).

## 2 Methods

### 2.1 Study design and reporting

**Figure 1:**
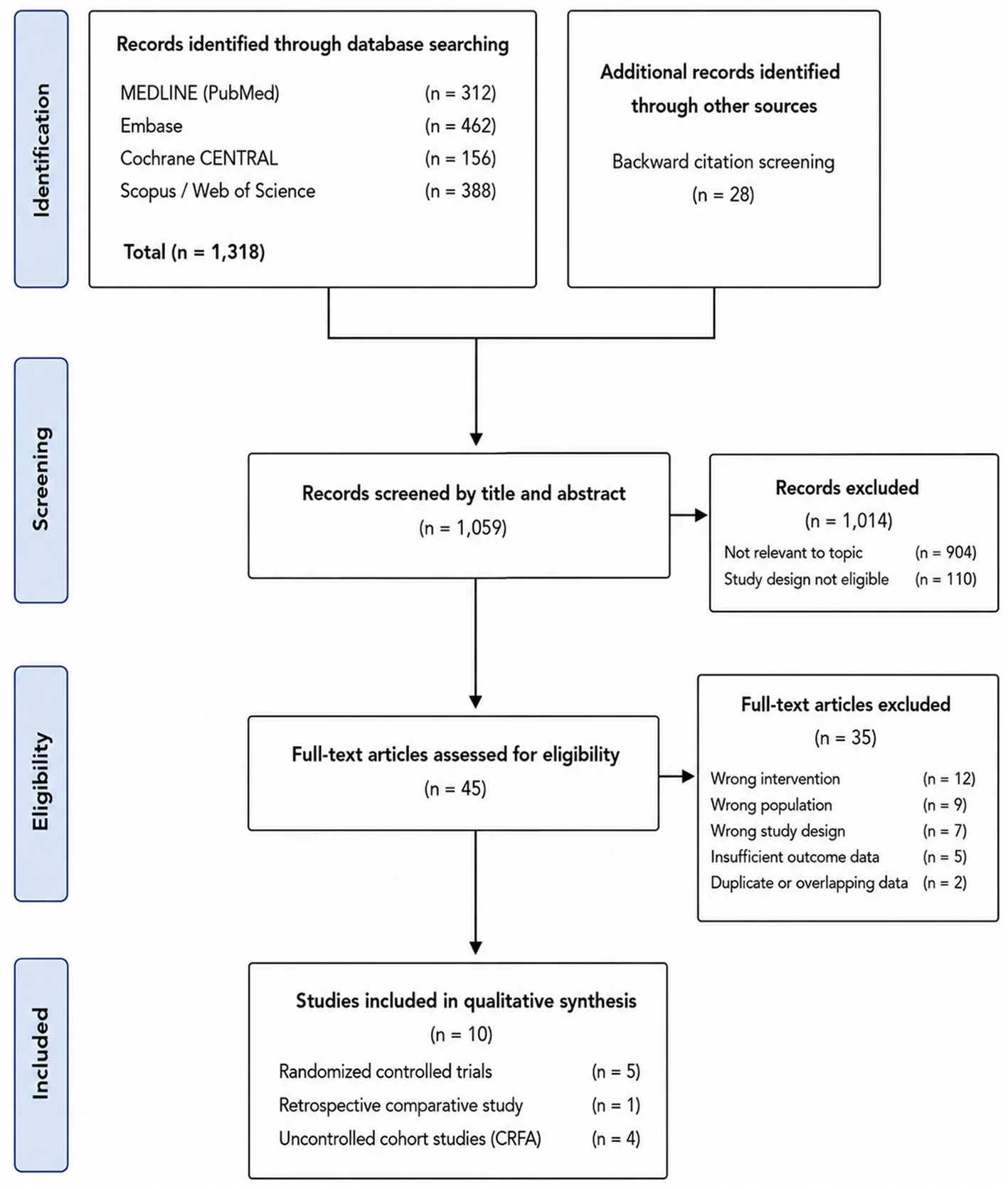
PRISMA 2020 flow diagram illustrating the study selection process for the systematic review.

This systematic review was conducted in accordance with the Preferred Reporting Items for Systematic Reviews and Meta-Analyses (PRISMA) 2020 statement (14). The review was registered in the International Prospective Register of Systematic Reviews (PROSPERO; Registration No. CRD420261295837). The study-selection process is illustrated in the PRISMA 2020 flow diagram (Figure 1).

### 2.2 Search strategy and information sources

A comprehensive literature search was conducted in MEDLINE (via PubMed), Embase, the Cochrane Central Register of Controlled Trials (CENTRAL), Scopus, and Web of Science from database inception to 30 July 2026. The search strategy combined controlled vocabulary (where applicable) and free-text terms related to chronic shoulder pain, pulsed radiofrequency (PRF), cooled radiofrequency ablation (CRFA), suprascapular nerve, and shoulder denervation. In addition, backward citation screening of the reference lists of eligible studies and relevant systematic reviews was performed to identify additional potentially eligible articles. No restrictions were applied regarding publication year or language.

### 2.3 Eligibility criteria

Eligibility criteria were defined according to the Population, Intervention, Comparator, Outcomes, and Study Design (PICOS) framework. The review included studies involving adults (≥18 years) with chronic shoulder pain. Interventions of interest were pulsed radiofrequency (PRF) or cooled radiofrequency ablation (CRFA) targeting the sensory innervation of the shoulder. Comparative PRF studies were necessary to include corticosteroid-based injections or active nerve blocks as comparators. For adjunctive PRF studies, the comparator included the same corticosteroid-based treatment without PRF. Uncontrolled CRFA cohort studies were eligible without a comparator and were synthesized separately. Outcomes of interest included pain intensity, shoulder-specific function, the range of motion, responder rates, the duration of pain relief, analgesic use, and procedure-related adverse events. Randomized controlled trials and prospective or retrospective observational studies were included. Case reports, conference abstracts, narrative reviews, systematic reviews, studies evaluating conventional thermal radiofrequency without separately reported PRF or CRFA outcomes, and studies comparing different PRF parameter settings without a clinically distinct comparator were excluded.

### 2.4 Study selection and data extraction

Titles, abstracts, and full-text articles were screened by the reviewer against the predefined eligibility criteria. Data extracted from each included study comprised study design, sample size, diagnosis, intervention characteristics, target nerves, comparator, follow-up duration, pain and functional outcomes, responder definitions, analgesic use, and procedure-related adverse events. Systematic reviews were used for citation screening and contextual interpretation but were not included in the qualitative synthesis. Study selection and data extraction were performed by a single reviewer and were not conducted independently in duplicate.

### 2.5 Methodological quality, risk of bias, and certainty of evidence

Risk of bias in randomized controlled trials was assessed using the Cochrane Risk of Bias 2 (RoB 2) tool (15), whereas the retrospective comparative study was evaluated using the Risk Of Bias In Non-randomized Studies of Interventions (ROBINS-I) tool (16). As the evidence for cooled radiofrequency ablation (CRFA) consisted of uncontrolled cohort studies, methodological quality was assessed using the Joanna Briggs Institute (JBI) Critical Appraisal Checklist for Case Series (17). Study-level methodological quality and risk-of-bias assessments are summarized descriptively. The certainty of evidence for each comparison was evaluated using the Grading of Recommendations, Assessment, Development and Evaluation (GRADE) approach (18), considering the domains of risk of bias, inconsistency, indirectness, imprecision, and publication bias. The certainty-of-evidence assessments were summarized narratively for each comparison.

### 2.6 Data synthesis

A quantitative meta-analysis was not performed because of substantial clinical and methodological heterogeneity across the included studies, including differences in patient populations, underlying shoulder disorders, radiofrequency targets, procedural techniques, comparator interventions, outcome measures, and follow-up duration. The findings were therefore synthesized narratively, with a separate synthesis of comparative PRF studies, adjunctive PRF studies, and uncontrolled CRFA cohort studies.

## 3 Results

### 3.1 Study selection

The study-selection process is summarized in Figure 1. A total of 45 full-text articles were assessed for eligibility, of which 35 were excluded for the following reasons: wrong intervention (*n* = 12), wrong population (*n* = 9), wrong study design (*n* = 7), insufficient outcome data (*n* = 5), and duplicate or overlapping data (*n* = 2). Ultimately, 10 primary studies met the eligibility criteria and were included in the qualitative synthesis, comprising five randomized controlled trials, one retrospective comparative study, and four uncontrolled cohort studies evaluating cooled radiofrequency ablation (CRFA).

Among the included studies, six evaluated pulsed radiofrequency (PRF) and four evaluated CRFA. Of the PRF studies, four randomized controlled trials compared PRF with active comparator interventions, whereas two studies evaluated PRF as an adjunct to corticosteroid-based treatment. All evidence for CRFA was derived from uncontrolled cohort studies.

### 3.2 Characteristics of the included studies

The 10 included studies enrolled 476 participants with chronic shoulder pain associated with adhesive capsulitis, post-stroke hemiplegic shoulder pain, shoulder impingement syndrome, glenohumeral osteoarthritis, rotator cuff-related disorders, and mixed chronic shoulder pain. In total, six studies investigated pulsed radiofrequency (PRF), including five randomized controlled trials and one retrospective comparative study, whereas four uncontrolled cohort studies evaluated cooled radiofrequency ablation (CRFA). Follow-up durations ranged from 1 to 12 months. The characteristics of the included studies are summarized in Table 1.

**Table 1:** Characteristics of the included studies.

Study	Design (*n*)	Population	Intervention	Comparator	Follow-up	Main findings
Eyigor et al. (19)	RCT (50)	Painful shoulder	Suprascapular PRF	Intra-articular corticosteroid	12 weeks	Significant improvements occurred in both groups. Intra-articular corticosteroid injection resulted in greater early improvements in pain intensity, SPADI scores, and analgesic use.
Kim and Chang (20)	RCT (20)	Post-stroke hemiplegic shoulder pain	Suprascapular PRF	Intra-articular corticosteroid	2 months	Significant improvements occurred in both groups. Intra-articular corticosteroid injection resulted in greater reductions in pain intensity and greater improvements in the shoulder range of motion.
Gupta et al. (21)	RCT (40)	Chronic shoulder pain	US-guided suprascapular PRF	Suprascapular nerve block (local anesthetic + corticosteroid)	12 weeks	Significant improvements occurred in both groups. Apart from lower pain scores in the corticosteroid group at 2 weeks, no significant between-group differences were observed at the primary 12-week endpoint or for functional outcomes.
Bergamaschi et al. (22)	RCT (40)	Chronic shoulder pain	Suprascapular PRF	US-guided suprascapular nerve block	12 weeks	PRF resulted in greater reductions in movement pain at 2–8 weeks and resting pain at 12 weeks, whereas shoulder range of motion was comparable between groups.
Abdelfatah Sayed et al. (23)	RCT (60)	Shoulder impingement syndrome	PRF + SSN block + glenohumeral corticosteroid	SSN block + glenohumeral corticosteroid	6 months	Adjunctive PRF resulted in greater reductions in pain intensity and SPADI scores together with greater improvements in active shoulder range of motion.
Çetingök and Serçe (24)	Retrospective comparative (160)	Chronic shoulder pain	PRF + SSN block + intra-articular corticosteroid	SSN block + intra-articular corticosteroid	1 months^^	Adjunctive PRF was associated with lower pain intensity, longer median pain relief (6 vs. 4 months), and higher patient satisfaction.
Tran et al. (25)	Prospective pilot (12)	Glenohumeral osteoarthritis	CRFA (axillary, lateral pectoral, suprascapular nerves)	None	6 months	CRFA was associated with marked reductions in pain and improvements in shoulder function, with VAS scores decreasing from 8.8 to 2.2 and ASES scores increasing from 17.2 to 65.7; no major complications were reported.
Santi et al. (26)	Retrospective cohort (44)	Chronic shoulder pain	CRFA (articular branches)	None	12 months	CRFA provided sustained pain relief and functional improvement, with ≥50% pain relief in 70.4%, 61.0%, and 51.0% of patients at 1, 6, and 12 months, respectively; OSS improved, and analgesic use decreased.
Yoshida et al. (27)	Prospective cohort (35)	Mixed chronic shoulder disorders	Internally cooled multi-nerve CRFA	None	6 months	CRFA was associated with sustained improvements in pain intensity, ASES scores, and active shoulder flexion and abduction throughout follow-up.
Nava-Obregon et al. (28)	Prospective cohort (15)	Chronic shoulder pain	Multi-nerve CRFA	None	24 weeks	CRFA was associated with improvements in pain intensity and shoulder function throughout the 24-week follow-up period.

Follow-up timing was reported according to the assessment schedule in the original publication. †1-month outcome assessment with additional reporting of the duration of pain relief.

ASES, American Shoulder and Elbow Surgeons score; CRFA, cooled radiofrequency ablation; NRS, numeric rating scale; OSS, Oxford Shoulder Score; PRF, pulsed radiofrequency; ROM, range of motion; SPADI, Shoulder Pain and Disability Index; SSN, suprascapular nerve; US, ultrasound; VAS, visual analog scale.

### 3.3 PRF compared with corticosteroid-based or active nerve block interventions

In total, four randomized controlled trials evaluated PRF against corticosteroid-based injections or active suprascapular nerve block interventions. All studies demonstrated significant improvements in pain intensity and shoulder function following PRF. However, PRF did not demonstrate superiority over intra-articular corticosteroid injection. In addition, two randomized trials favored corticosteroid injection for short-term pain reduction and shoulder mobility, one trial reported comparable outcomes at the primary 12-week endpoint, except for earlier pain relief favoring corticosteroid injection, and one trial demonstrated superior pain outcomes with PRF compared to a local-anesthetic suprascapular nerve block. Collectively, randomized evidence demonstrates that PRF consistently improves pain and shoulder function but does not establish superiority over corticosteroid-based interventions, which generally provide greater short-term pain relief.

### 3.4 Adjunctive PRF

Unlike the comparative PRF trials, two studies involving 220 participants evaluated PRF as an adjunct to corticosteroid-based treatment rather than as an alternative intervention (23, 24). In the randomized trial by Abdelfatah Sayed et al., the addition of PRF to a suprascapular nerve block and glenohumeral corticosteroid injection resulted in lower pain intensity, lower Shoulder Pain and Disability Index (SPADI) scores, and greater improvements in several active range-of-motion measures over 6 months of follow-up (23). Similarly, Çetingök and Serçe retrospectively compared patients receiving glenohumeral intra-articular corticosteroid injection and a suprascapular nerve block with or without adjunctive PRF. Baseline pain scores were comparable, whereas adjunctive PRF was associated with lower 1-month pain scores, longer median pain relief (6 months vs. 4 months), and higher patient satisfaction (24). Overall, both studies suggest that adjunctive PRF may enhance clinical outcomes when combined with corticosteroid-based treatment; however, because PRF was delivered as part of multimodal treatment packages, its independent contribution cannot be determined.

### 3.5 Cooled radiofrequency ablation

In total, four uncontrolled cohort studies involving 106 participants evaluated cooled radiofrequency ablation (CRFA) for chronic shoulder pain (25–28). Across studies, CRFA was associated with reductions in pain intensity and improvements in shoulder function during follow-up. Tran et al. reported reductions in visual analog scale (VAS) pain scores from 8.8 at baseline to 2.2 at 6 months, accompanied by an increase in American Shoulder and Elbow Surgeons (ASES) scores from 17.2 to 65.7 in patients with glenohumeral osteoarthritis (25). Santi et al. reported that 70.4%, 61.0%, and 51.0% of patients achieved at least 50% pain relief at 1, 6, and 12 months, respectively, along with improvements in the Oxford Shoulder Score and reduced analgesic use (26). Yoshida et al. observed improvements in pain intensity, ASES scores, and active shoulder flexion and abduction over 6 months following internally cooled multi-nerve CRFA (27). Nava-Obregon et al. also reported improvements in pain and functional outcomes during the 24-week follow-up period (28). Although these findings suggest sustained clinical benefit in selected patients, the absence of concurrent control groups limits comparative interpretation. All CRFA studies selected patients using positive diagnostic blocks before denervation, potentially enriching study populations for treatment responders and limiting generalizability to unselected patients.

### 3.6 Functional outcomes

Functional outcomes generally followed the pattern observed for pain intensity. Comparative PRF studies reported improvements in shoulder-specific function, including the Shoulder Pain and Disability Index (SPADI) and range-of-motion measures, although between-group differences varied across studies (19–24). Similarly, uncontrolled CRFA cohort studies consistently reported improvements in shoulder function using validated instruments, including the American Shoulder and Elbow Surgeons (ASES) score and Oxford Shoulder Score, together with improvements in the range of motion of the shoulder where assessed (25–28). Direct comparison of functional outcomes across studies was limited by differences in underlying diagnoses, outcome measures, and follow-up schedules.

### 3.7 Safety

No serious procedure-related adverse events were reported across the included studies (Table 2). Reported adverse events were infrequent and generally transient. Bergamaschi et al. reported local pain and one hematoma following PRF, whereas the nerve block group experienced one hematoma and two episodes of lipothymia (22). Tran et al. reported no major complications following CRFA (25), and Abdelfatah Sayed et al. reported no treatment-related complications during the 6-month follow-up period (23). Adverse event reporting was limited in several studies; therefore, the available evidence does not allow definitive conclusions regarding the safety profile of PRF or CRFA. In addition, the overall sample size was insufficient to reliably assess uncommon complications, particularly following multi-nerve CRFA.

**Table 2:** Reported safety outcomes of the included studies.

Study	Intervention	Reported adverse events	Comments
Eyigor et al. (19)	PRF	No serious procedure-related adverse events reported.	Safety reporting was limited by the small sample size.
Kim and Chang (20)	PRF	No major complications reported.	No detailed safety analysis was provided.
Gupta et al. (21)	PRF	No serious procedure-related adverse events reported.	Adverse event reporting was limited.
Bergamaschi et al. (22)	PRF	Local pain (*n* = 1) and hematoma (*n* = 1); comparator group: hematoma (*n* = 1) and lipothymia (*n* = 2).	All reported adverse events were transient and resolved without major sequelae.
Abdelfatah Sayed et al. (23)	Adjunctive PRF	No treatment-related adverse events during the 6-month follow-up.	Prospective safety assessment.
Çetingök and Serçe (24)	Adjunctive PRF	No detailed adverse event data reported.	Retrospective study with limited safety reporting.
Tran et al. (25)	CRFA	No major complications reported.	Prospective pilot study (*n* = 12).
Santi et al. (26)	CRFA	No major procedure-related adverse events reported.	Retrospective cohort study.
Yoshida et al. (27)	CRFA	No major complications reported.	Prospective cohort study.
Nava-Obregon et al. (28)	CRFA	No major complications reported.	Prospective cohort study (*n* = 15).

CRFA, cooled radiofrequency ablation; PRF, pulsed radiofrequency.

### 3.8 Methodological quality and certainty of evidence

**Figure 2:**
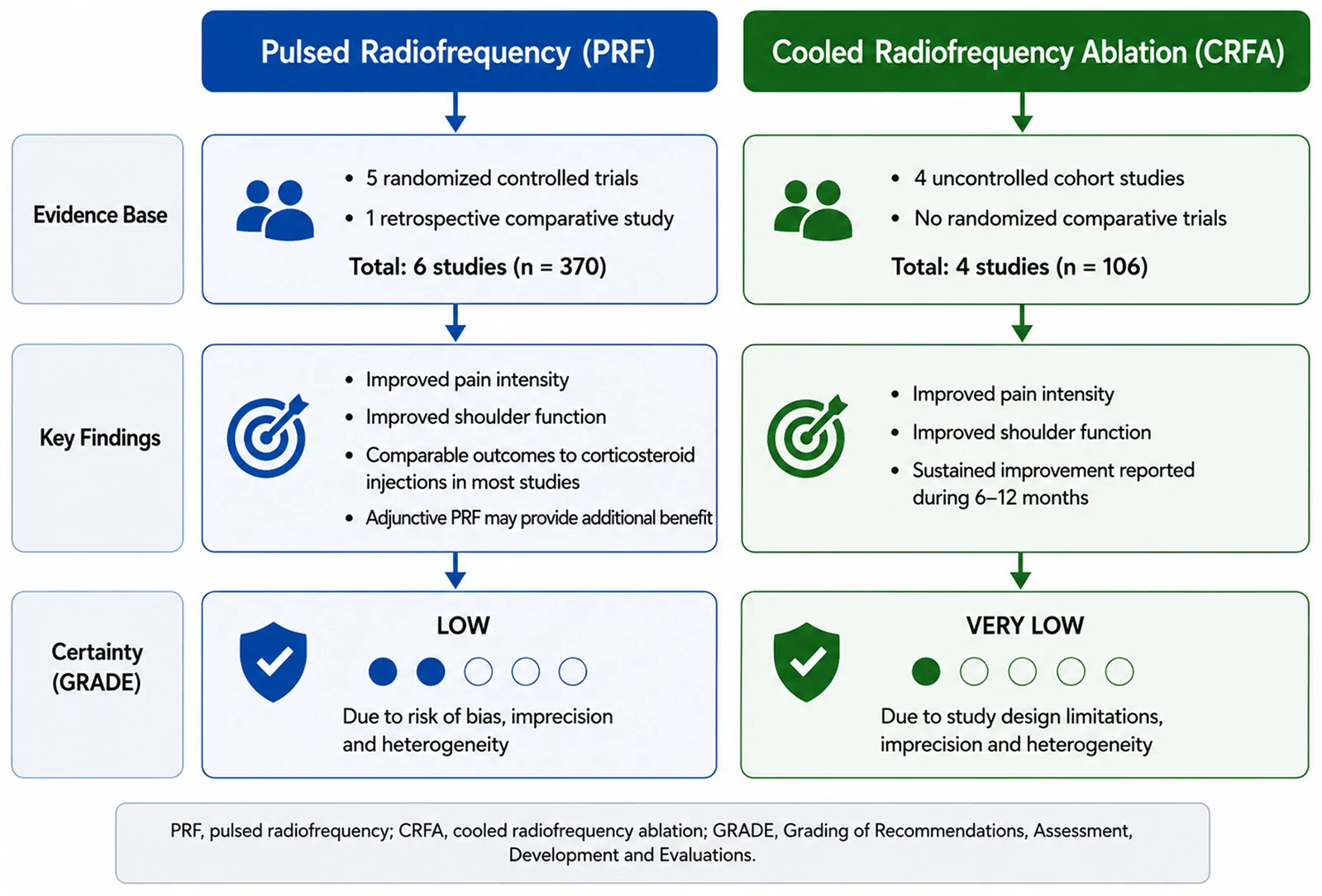
Overview of the current evidence supporting PRF and CRFA for chronic shoulder pain.

Methodological quality and risk of bias varied across the included studies (Table 3). Randomized controlled trials were judged as having some concerns using the Cochrane Risk of Bias 2 (RoB 2) tool, primarily because of incomplete reporting of allocation procedures, limited blinding, small sample sizes, and reliance on patient-reported outcomes. The retrospective comparative study was judged to be at serious risk of bias using ROBINS-I because of non-randomized treatment allocation and the potential for confounding. The uncontrolled CRFA cohort studies were appraised using the Joanna Briggs Institute (JBI) Critical Appraisal Checklist for Case Series; as no prespecified numerical threshold was used, no overall categorical quality rating was assigned. Their principal limitations were the absence of concurrent control groups, small samples in several studies, and the inherent limitations of uncontrolled observational designs. According to the GRADE approach, the certainty of evidence was rated as low for comparative PRF studies, low for adjunctive PRF studies, and very low for uncontrolled CRFA cohort studies. The narrative summary of findings and certainty assessments is presented in Table 4, while an overview of the current evidence supporting PRF and CRFA for chronic shoulder pain is provided in Figure 2.

**Table 3:** Methodological quality and risk-of-bias assessment of the included studies.

Study	study design	Assessment tool	Overall assessment	Key methodological limitations
Eyigor et al. (19)	Randomized controlled trial	RoB 2	Some concerns	Incomplete reporting of allocation concealment and blinding; reliance on patient-reported outcomes.
Kim and Chang (20)	Randomized controlled trial	RoB 2	Some concerns	Small sample size, limited blinding, and subjective outcome assessment.
Gupta et al. (21)	Randomized controlled trial	RoB 2	Some concerns	Limited reporting of randomization and blinding procedures.
Bergamaschi et al. (22)	Randomized controlled trial	RoB 2	Some concerns	Limited blinding and reliance on patient-reported pain outcomes.
Abdelfatah Sayed et al. (23)	Randomized controlled trial	RoB 2	Some concerns	Modest sample size and incomplete reporting of blinding procedures.
Çetingök and Serçe (24)	Retrospective comparative study	ROBINS-I	Serious risk of bias	Non-randomized treatment allocation, potential confounding, and retrospective outcome assessment.
Tran et al. (25)	Prospective pilot cohort	JBI case series checklist	No overall category assigned	Small sample size, uncontrolled design, and absence of a concurrent comparator.
Santi et al. (26)	Retrospective cohort	JBI case series checklist	No overall category assigned	Retrospective design without a concurrent control group.
Yoshida et al. (27)	Prospective cohort	JBI case series checklist	No overall category assigned	Uncontrolled design and participant selection following positive diagnostic blocks.
Nava-Obregon et al. (28)	Prospective cohort	JBI case series checklist	No overall category assigned	Small sample size, uncontrolled design, and absence of a concurrent comparator.

RoB 2, cochrane risk of bias 2 tool; ROBINS-I, risk of bias in non-randomized studies of interventions; JBI, Joanna Briggs Institute Critical Appraisal Checklist for Case Series.

**Table 4:** Narrative summary of the findings and certainty of evidence (GRADE).

Comparison	Studies (participants)	Pain	Function	Safety	GRADE certainty	Clinical interpretation
PRF vs. corticosteroid-based or active nerve block interventions	Four RCTs (*n* = 150)	Improved in both groups; no consistent between-group advantage	Improved in both groups; functional differences were inconsistent	No major procedure-related adverse events reported	Low	PRF improved pain and shoulder function but did not demonstrate superiority over corticosteroid-based interventions, which generally provide greater short-term pain relief.
Adjunctive PRF + corticosteroid-based treatment	One RCT + 1 retrospective comparative study (*n* = 220)	Greater reductions in pain intensity	Greater functional improvement and/or longer pain relief	No major complications reported; safety reporting varied	Low	Adjunctive PRF may provide incremental clinical benefit, although evidence remains limited.
CRFA	Four cohort studies (*n* = 106)	Sustained improvement during follow-up	Shoulder function consistently improved	No major complications reported in small cohorts	Very low	CRFA demonstrated favorable observational outcomes, but comparative effectiveness remains uncertain.

GRADE considerations: The certainty of evidence for comparative PRF studies was rated as low because of concerns regarding risk of bias and inconsistency across randomized trials. Evidence for adjunctive PRF studies was also rated as low, owing to the risk of bias and imprecision associated with the limited number of available studies. The certainty of evidence for CRFA studies was rated as very low because it was derived entirely from uncontrolled observational studies with serious risk of bias, substantial imprecision, and no comparative evidence.

GRADE certainty levels: high, very confident that the true effect is close to the estimate; moderate, moderately confident; low, limited confidence; and very low, very little confidence in the estimated effect. Certainty was assessed across the domains of risk of bias, inconsistency, indirectness, imprecision, and publication bias.

## 4 Discussion

### 4.1 Principal findings

This systematic review synthesized the available evidence on pulsed radiofrequency (PRF) and cooled radiofrequency ablation (CRFA) for chronic shoulder pain by separately evaluating comparative PRF studies, adjunctive PRF studies, and uncontrolled CRFA cohort studies. Randomized evidence consistently demonstrated that PRF improved pain intensity and shoulder function in patients with chronic shoulder pain. However, superiority over corticosteroid-based interventions was not established. Among the four randomized comparative studies, two favored intra-articular corticosteroid injection for short-term pain reduction and shoulder mobility, one reported comparable outcomes at the primary endpoint except for earlier pain relief favoring corticosteroid injection, and one demonstrated superior pain outcomes with PRF compared with a local-anesthetic suprascapular nerve block. Overall, these findings support PRF as an effective intervention but do not demonstrate consistent superiority over corticosteroid-based treatment. These observations are consistent with a recent systematic review and meta-analysis, which also identified substantial heterogeneity in patient populations, procedural techniques, and outcome assessments across PRF studies (29).

The evidence for adjunctive PRF was more consistent. Both the randomized trial and the retrospective comparative study reported greater or more durable clinical benefit when PRF was added to corticosteroid-based treatment than when corticosteroid-based treatment was administered alone. Although these findings suggest that PRF may enhance and prolong treatment response, both studies evaluated multimodal treatment packages, preventing determination of the independent contribution of PRF.

The evidence supporting CRFA showed a consistent direction of improvement in pain intensity and shoulder function across all included cohort studies. Nevertheless, all available CRFA studies were observational, lacked concurrent control groups, and selected patients after positive diagnostic blocks, limiting both causal inference and generalizability. Consequently, the apparent consistency of CRFA outcomes should be interpreted in the context of the lower methodological quality and very low certainty of the available evidence.

Taken together, current evidence suggests that radiofrequency-based interventions may represent useful treatment options for carefully selected patients with chronic shoulder pain that persists despite conservative management. Comparative evidence supporting PRF is stronger than that supporting CRFA, whereas evidence for adjunctive PRF indicates a potential incremental benefit when combined with corticosteroid-based treatment. However, the current evidence does not establish a preferred radiofrequency strategy, and adequately powered randomized comparative trials are required before definitive treatment recommendations can be made. The low certainty ratings for comparative and adjunctive PRF studies and the very low certainty rating for CRFA studies indicate that future well-designed randomized studies are likely to influence the estimated treatment effects and may change the current conclusions.

### 4.2 Interpretation of comparative PRF findings

The comparative PRF studies demonstrated that treatment effects varied according to the clinical setting and active comparator rather than showing a consistent advantage for either intervention. Differences in underlying shoulder pathology, comparator composition, PRF technique, follow-up duration, and outcome assessment likely contributed to the observed variability. Randomized trials comparing PRF with intra-articular corticosteroid injection generally favored corticosteroid treatment for short-term pain relief and, in some studies, shoulder mobility, whereas the trial comparing PRF with a local-anesthetic suprascapular nerve block demonstrated superior pain outcomes with PRF (19–22).

These findings suggest that the apparent comparative effectiveness of PRF depends on both the comparator intervention and the timing of assessment rather than reflecting an intrinsic treatment-specific advantage. Corticosteroid injections provide potent early anti-inflammatory effects, which may explain their superior short-term analgesic effects, whereas PRF is intended to modulate nociceptive transmission without causing neural destruction. Whether these differing mechanisms translate into distinct long-term clinical benefits remains uncertain because follow-up durations were relatively short and sample sizes were modest. Consequently, current randomized evidence supports PRF as an effective intervention but does not establish superiority over corticosteroid-based treatment.

### 4.3 PRF as an adjunct to corticosteroid-based treatment

Unlike the comparative PRF studies, the available adjunctive PRF studies evaluated whether adding PRF to corticosteroid-based treatment provides additional clinical benefit rather than whether PRF can replace corticosteroid injection. Both the randomized trial and the retrospective comparative study consistently reported greater reductions in pain, longer duration of analgesia, or improved functional recovery when PRF was combined with corticosteroid-based treatment than when corticosteroid-based treatment was administered alone (23, 24). This consistency across two different study designs supports the hypothesis that PRF may have an additive therapeutic role.

One possible explanation is that corticosteroid injections and PRF target different mechanisms contributing to chronic shoulder pain. Corticosteroids primarily reduce inflammation, whereas PRF has been proposed to alter nociceptive signaling through neuromodulatory mechanisms. Consequently, combining these interventions may offer both early anti-inflammatory effects and more prolonged modulation of pain transmission. However, because both studies evaluated multimodal treatment packages that also included a suprascapular nerve block, local anesthetic, and rehabilitation, the independent contribution of PRF cannot be isolated. Accordingly, the observed benefits should be interpreted as evidence supporting a multimodal treatment strategy rather than PRF alone.

Future adequately powered randomized trials comparing PRF alone, corticosteroid-based treatment alone, and combination therapy are required to determine whether adjunctive PRF provides clinically meaningful incremental benefit beyond established injection-based treatment.

### 4.4 Interpretation of CRFA findings

The available evidence suggests that cooled radiofrequency ablation (CRFA) may lead to sustained improvements in pain intensity and shoulder function in carefully selected patients with chronic shoulder pain. However, all currently available evidence originates from uncontrolled observational studies, preventing the reliable estimation of comparative treatment effects or the determination of superiority over pulsed radiofrequency (PRF) or corticosteroid-based interventions.

Unlike PRF, CRFA intentionally produces thermal neuroablation, while internal electrode cooling permits delivery of radiofrequency energy over a larger treatment volume. This technique may improve lesion coverage despite the anatomical variability of shoulder sensory innervation. Cadaveric investigations have demonstrated that the sensory innervation of the glenohumeral joint is distributed across the suprascapular, axillary, lateral pectoral, and other terminal articular branches, providing the anatomical rationale for contemporary multi-nerve denervation strategies (9, 10). More recent procedural refinements have proposed expanded targeting of terminal sensory branches to further improve lesion coverage; however, these technical modifications have not yet been validated in comparative clinical studies (30).

Interpretation of the current CRFA literature also requires consideration of patient selection. A majority of cohorts required a positive diagnostic block before ablation, thereby enriching study populations with patients considered more likely to respond to sensory denervation. Although this approach reflects current interventional practice and may improve procedural success, it limits generalizability to broader populations with chronic shoulder pain.

The absence of randomized or controlled comparative studies remains the principal limitation of the current CRFA literature. Improvements observed after treatment may reflect the procedural effect, regression to the mean, placebo response, patient selection, concurrent rehabilitation, changes in analgesic therapy, or the natural history of symptoms. Consequently, although the available evidence supports CRFA as a promising option for carefully selected patients with refractory chronic shoulder pain, its comparative effectiveness and optimal place within the interventional treatment pathway remain uncertain. Well-designed randomized controlled trials are required before definitive recommendations can be made.

### 4.5 Clinical implications

The available evidence suggests that radiofrequency-based interventions may be considered for carefully selected patients with chronic shoulder pain who remain symptomatic despite appropriate conservative management. Treatment selection should be individualized through shared decision-making, taking into account the underlying shoulder pathology, previous treatment response, functional goals, patient preferences, contraindications to corticosteroid therapy, procedural expertise, and available resources.

Randomized comparative evidence suggests that PRF improves pain intensity and shoulder function but does not demonstrate superiority over corticosteroid-based interventions, which generally provide greater short-term pain relief (19–22, 29). Accordingly, PRF may be considered for patients in whom repeated corticosteroid injections are contraindicated, undesirable, or have failed to provide sustained benefit, particularly when a non-ablative intervention is preferred.

Adjunctive PRF represents a distinct therapeutic strategy. Preliminary evidence from one randomized trial and one retrospective comparative study suggests that combining PRF with corticosteroid-based treatment may enhance or prolong clinical benefit (23, 24). However, because both studies evaluated multimodal treatment packages, the independent contribution of PRF remains uncertain, and current evidence is insufficient to support its routine use.

The available evidence also suggests that CRFA may be considered for carefully selected patients with persistent chronic shoulder pain, particularly following a positive diagnostic block indicating that articular sensory nerves contribute substantially to symptoms (25–28). However, since all available evidence are derived from uncontrolled cohort studies, its comparative effectiveness relative to PRF or corticosteroid-based treatment remains uncertain.

Overall, current evidence does not support a fixed treatment hierarchy among corticosteroid injections, PRF, and CRFA. Instead, interventional management should be individualized and integrated with rehabilitation, with treatment decisions informed by the quality and certainty of the available evidence, anticipated clinical benefits, procedural risks, patient preferences, and shared decision-making between patients and clinicians.

### 4.6 Strengths

This review addressed a focused clinical question by separately synthesizing comparative PRF studies, adjunctive PRF studies, and uncontrolled CRFA cohort studies. By distinguishing these fundamentally different treatment strategies, the review avoided inappropriate pooling of studies with different clinical objectives, comparator interventions, procedural intent, and levels of evidence, allowing each evidence body to be interpreted within its appropriate clinical context.

Randomized and observational studies were synthesized separately, avoiding the direct comparison between study designs with different susceptibility to bias. Methodological quality and risk of bias were evaluated using study design-specific appraisal tools, while the certainty of evidence was assessed using the GRADE approach, providing a structured and transparent interpretation of the available literature. The review was conducted according to the PRISMA 2020 reporting guideline and incorporated a comprehensive search of multiple bibliographic databases, together with backward citation screening, thereby further strengthening the methodological rigor and reproducibility of the review.

### 4.7 Limitations

Several limitations should be considered when interpreting the findings of this review. Although a comprehensive search of multiple bibliographic databases was performed, the available evidence remained limited by the small number of eligible studies and the methodological quality of the included literature. Comparative PRF studies generally enrolled modest sample sizes, whereas evidence supporting CRFA was derived entirely from uncontrolled cohort studies, resulting in substantially lower certainty of evidence for CRFA than for PRF.

Considerable clinical and methodological heterogeneity further limited the interpretation of the findings. The included studies evaluated diverse shoulder disorders, including post-stroke hemiplegic shoulder pain, shoulder impingement syndrome, glenohumeral osteoarthritis, and mixed chronic shoulder pain populations. Differences in patient characteristics, radiofrequency targets, procedural techniques, comparator interventions, outcome measures, and follow-up duration precluded a quantitative meta-analysis and limited direct comparison across studies.

No eligible study directly compared PRF with CRFA. Consequently, differences in reported clinical outcomes between these interventions represent indirect comparisons across separate study populations and study designs rather than evidence of comparative effectiveness or superiority.

Although no serious procedure-related adverse event signal was identified, safety findings should be interpreted cautiously because adverse event reporting was inconsistent across studies and the cumulative sample size was insufficient to reliably detect uncommon complications.

Finally, study selection and data extraction were performed by a single reviewer without independent duplicate assessment, introducing the possibility of selection and data extraction errors despite the use of predefined eligibility criteria.

### 4.8 Future directions

The highest research priority is a sufficiently powered, multicenter randomized controlled trial directly comparing PRF, CRFA, and a clinically relevant corticosteroid-based intervention. Standardization of diagnostic criteria, patient selection, anatomical targets, procedural parameters, diagnostic block protocols, rehabilitation strategies, outcome measures, responder definitions, and follow-up schedules would substantially improve comparability across future studies.

Adjunctive PRF should be evaluated independently using randomized designs comparing PRF alone, corticosteroid-based treatment alone, and combination therapy. Such studies are required to determine whether PRF provides a clinically meaningful incremental benefit beyond established injection-based treatment.

Future CRFA studies should incorporate active comparator groups, prespecified responder criteria, and standardized procedural reporting. Stratification according to the underlying shoulder pathology and the response to diagnostic blocks may help identify patients most likely to benefit from articular sensory denervation while improving the external validity of future studies.

Long-term follow-up is needed to determine the durability of treatment response, recurrence, repeat procedures, and cumulative safety. Future trials should also incorporate patient-centered outcomes, including quality of life, sleep, analgesic consumption, return to work or usual activities, patient satisfaction, healthcare utilization, and cost-effectiveness, thereby providing a more comprehensive evaluation of the clinical value of radiofrequency-based interventions.

## 5 Conclusion

This systematic review found that both pulsed radiofrequency (PRF) and cooled radiofrequency ablation (CRFA) were associated with improvements in pain intensity and shoulder function in patients with chronic shoulder pain. Randomized evidence suggests that PRF improves pain intensity and shoulder function; however, it has not demonstrated superiority over corticosteroid-based injections, which generally provide greater short-term pain relief. Preliminary evidence suggests that adjunctive PRF may enhance or prolong clinical benefit when combined with corticosteroid-based treatment, although the independent contribution of PRF remains uncertain. Evidence supporting CRFA is currently limited to uncontrolled observational studies, precluding a reliable assessment of its comparative effectiveness.

Radiofrequency-based interventions may be considered part of an individualized management strategy for carefully selected patients with persistent chronic shoulder pain that has not responded adequately to conservative treatment. However, the certainty of evidence remains low for comparative and adjunctive PRF studies and very low for CRFA studies. Adequately powered, multicenter randomized controlled trials directly comparing PRF, CRFA, and corticosteroid-based interventions are required before a preferred radiofrequency strategy or treatment hierarchy can be recommended.

## Data Availability

The original contributions presented in the study are included in the article/supplementary material, further inquiries can be directed to the corresponding author.
